# Host-directed therapy for bacterial infections -Modulation of the phagolysosome pathway-

**DOI:** 10.3389/fimmu.2023.1227467

**Published:** 2023-09-29

**Authors:** Toshihiko Taya, Fumiya Teruyama, Satoshi Gojo

**Affiliations:** ^1^ Department of Cardiovascular Medicine, Graduate School of Medicine, Kyoto Prefectural University of Medicine, Kyoto, Japan; ^2^ Pharmacology Research Department, Tokyo New Drug Research Laboratories, Kowa Company, Ltd., Tokyo, Japan; ^3^ Department of Regenerative Medicine, Graduate School of Medicine, Kyoto Prefectural University of Medicine, Kyoto, Japan

**Keywords:** bacterial infection, immune evasion, host-directed therapy, sepsis, antimicrobial resistance, phagocytosis, lysosome, V-ATPase

## Abstract

Bacterial infections still impose a significant burden on humanity, even though antimicrobial agents have long since been developed. In addition to individual severe infections, the f fatality rate of sepsis remains high, and the threat of antimicrobial-resistant bacteria grows with time, putting us at inferiority. Although tremendous resources have been devoted to the development of antimicrobial agents, we have yet to recover from the lost ground we have been driven into. Looking back at the evolution of treatment for cancer, which, like infectious diseases, has the similarity that host immunity eliminates the lesion, the development of drugs to eliminate the tumor itself has shifted from a single-minded focus on drug development to the establishment of a treatment strategy in which the de-suppression of host immunity is another pillar of treatment. In infectious diseases, on the other hand, the development of therapies that strengthen and support the immune system has only just begun. Among innate immunity, the first line of defense that bacteria encounter after invading the host, the molecular mechanisms of the phagolysosome pathway, which begins with phagocytosis to fusion with lysosome, have been elucidated in detail. Bacteria have a large number of strategies to escape and survive the pathway. Although the full picture is still unfathomable, the molecular mechanisms have been elucidated for some of them, providing sufficient clues for intervention. In this article, we review the host defense mechanisms and bacterial evasion mechanisms and discuss the possibility of host-directed therapy for bacterial infection by intervening in the phagolysosome pathway.

## 1 Introduction: infection and drug development

### 1.1 Sepsis

The treatment of bacterial infections has changed dramatically with the development of antibiotics, and many lives have been saved; in the era of the COVID-19 pandemic, tuberculosis, a widespread and deadly disease, has not been conquered, but it has been set aside as an infectious disease. On the other hand, bacterial infections are still strictly a major threat to people’s lives and a major treatment target, one of which is sepsis caused by a rapid and massive bacterial load (1) and another is the emergence of antimicrobial resistance bacteria spurred by antibiotic overuse (2). Infectious diseases other than sepsis, such as tuberculosis, infectious gastroenteritis, bacterial pneumonia, and food poisoning, are limited to localized areas, and dysfunction of the tissues of the infected foci comes to the fore. Sepsis is defined as organ damage due to an inadequate host response to infection (3). In addition to the classic treatment of infusion, removal of infected lesions, and respiratory circulatory support, treatment is aimed at normalizing coagulation abnormalities to maintain organ microcirculation (4). Nevertheless, more than 11 million lives are lost annually to sepsis, making it the cause of nearly 20% of deaths worldwide (5). One of the reasons that current medical treatment for sepsis has been so hampered is that the host’s immune system forms a troublesome response to sepsis. That response is the coexistence of an excessive inflammatory response and a prolonged state of immunosuppression (6). The former, also called a cytokine storm, is characterized by an overproduction of inflammatory cytokines as the predominant phenotype (7). Much of the pathology of sepsis is associated with this unhelpfully exuberant reaction of the host, which is thought to be a common end pathway that occurs with viral infections as well as bacterial infections, and suppression of excess cytokines and regulation of their receptors is thought to reduce the disease state (8). However, the major proinflammatory cytokines IL-1 (9) and TNF-α (10) which are major inflammatory cytokines in sepsis, have failed to improve the survival of sepsis. In addition, inhibitors of Toll-like receptor (TLR) 4, which detects many bacteria and transduces intracellular signals that trigger inflammation, have also failed to improve sepsis survival (8). Despite such disappointment, transcriptome analysis of leukocytes from patient blood in sepsis revealed that up to 80% of the pathways of cellular function are altered and that inflammatory and regulatory mechanisms are simultaneously driven in the first few hours after onset (11). The setbacks in these clinical trials and the genetic approach to pathophysiology have led to a major shift in our current understanding of the pathogenesis of sepsis, in which host immunity to sepsis is a conflict between attack and suppression, far from its original goal of eliminating pathogens (12). This understanding of the pathogenesis has led to a search for therapeutic strategies that achieve homeostasis of host immune function.

### 1.2 Antimicrobial resistant bacteria

For antibiotics, the invention of new drugs in the nearly 30 years since the 1940s, a golden age, has been a wonderful scientific breakthrough that has led to an overly optimistic fantasy that bacterial infections will cease to be a threat to humanity (2). However, in the half-century since the 1970s, only a few new classes of antibiotics have been invented, and in addition, we have been handicapped by the disastrous situation with multidrug-resistant bacteria. Despite advances in understanding the life cycles of bacteria and long-awaited advances in molecular biology and genetics, biology and medicine today are far behind the good old days of the past 30 years in terms of progress in the field of infectious disease treatment. In the past half century, mankind has not only used invented antibiotics in large quantities in medicine but also abused them in livestock in search of economic rewards (13). The result has been a situation in which bacteria that have acquired multidrug resistance, also known as superbugs, have become rampant. Tuberculosis remains an uncontrolled infectious disease worldwide, and is the leading cause of mortality among mono infectious diseases, with 1.4 million deaths per year (14). Currently, it is estimated that 1/4 of the earth’s population is infected with *Mycobacterium tuberculosis*, the majority of which is considered to be in the latent stage, but reactivation is a common occurrence (15). Although the current standard of care is to continue the four-drug combination for at least six months (16), reinfection cannon be completely prevented, and 18% of these infections are caused by multidrug-resistant organisms (14, 17). Modern medicine has devoted many resources, and with the struggles of the field, has managed to prevent the global spread of antibiotic-resistant bacteria from becoming a pandemic threat like COVID-19. Given that the doubling time of bacteria is in hours, the speed of their molecular evolution is tremendous. Considering that even if a new drug is invented, it takes a certain amount of time for its clinical development, this weasel-word is extremely at disadvantage for humans. Nevertheless, new antibiotics are being developed to win the battle, and in addition, methods to attenuate bacterial toxins and phage-based methods (18, 19).

### 1.3 Current microbicidal strategy

Understanding the pathogenesis of sepsis is directly linked to drug development, which is moving toward therapies that can eliminate pathogens while balancing the active and regulatory systems of the immune system (6). One of the molecular basis of sepsis is the transformation of the energy supply system of immune cells, and various compounds related to PGC1α, which activates mitochondrial biosynthesis, are being investigated for their efficacy in the treatment of sepsis (20). In the treatment of antibiotic-resistant bacteria, the use of immune checkpoint inhibitors that block inhibitory signals in T cells, TGF-β to activate T cells, M1-like macrophage adaptive transfer, and strategies such as the administration of gelsolin, an endogenous protein, to enhance the pathogen clearance of macrophages are beginning to be explored (5). Methodologies to intervene in host immunity and promote pathogen elimination are beginning to emerge in the form of specific methods and compounds.

In this review, we focus on therapeutic strategies for infectious diseases through intervention in the host rather than approaches to the pathogens themselves. In the field of cancer therapy, the development of drugs aimed at killing the cancer cells themselves and the intervention of host immunity have made remarkable progress (21). Although immunity plays a major role in infectious diseases, the host approach has been neglected to date. Defense against microorganisms is mediated by the effector mechanisms of innate and adaptive immunity. Innate immunity is mainly responsible for defense in the early stages of infection, whereas adaptive immunity, together with innate immunity, provides a stronger and more specific response, and establishes a sustained defense posture with immune memory (22). The balance between these host immune responses and the acquisition of microbial resistance determines whether infection is established (23). The initial response of the host to bacterial infection is recognition of the bacteria by cells possessing pattern recognition receptors, release of inflammatory cytokines such as IL-1β, TNF-α, and IL-6, vasodilation, and increased vascular permeability (24). This leads to the accumulation of leukocytes, mainly neutrophils, which are non-specialized phagocytes, and more phagocytes in the infected foci. These cells phagocytose extracellular bacteria and infected cells and serve as the first line of defense in bacterial clearance (25). Inflammatory cytokines activate adaptive immunity, leading to enhanced antibody production by B cells, and opsonized bacteria are subject to phagocytosis by phagocytes, while T cells produce a variety of cytokines, including IFN-γ, to enhance bacterial killing by phagocytosis (26). Antibodies, together with activated complement, cause bacterial neutralization and lysis and play a role in the host defense system. In the early stages of infection, phagocytosis plays a central role in bacterial killing. On the other hand, bacteria that cause intracellular infection ensure their survival and replication by disabling the phagolysosomal system, which is the executor of intracellular disinfection.

Mycobacterium tuberculosis, which causes intracellular infection, can cause delayed-type hypersensitivity and tissue damage. Slow-growing *Mycobacterium tuberculosis* evades the killing of the phagolysosomal system and survives intracellularly, resulting in persistent stimulation of T cells and macrophages and the formation of granulomas (27). This granuloma and the solid tumor microenvironment share common features of immunosuppressive conditions such as lymphocyte exhaustion/elimination, macrophage polarization to M2-like phenotype, hypoxia, immunomodulatory cytokines such as TGF-β/IL-10, and infiltration of myeloid-derived suppressor cells (5). This similarity reminds us that host-directed therapy, which has been successful in anticancer therapy, could bear great fruit in infectious diseases. Among host immune mechanisms, the phagolysosomal system is considered to be at the center of pathogen control and an appropriate target for infection control. In order to examine the possibility of intervention in the phagolysosome system in host-directed therapy, the molecular mechanism of the pathway from phagocytosis to phagosomes reaching lysosomes is discussed from the perspective of host-pathogen interaction. Finally, the current status and future potential of drug discovery targeting the phagolysosome pathway will be discussed.

## 2 Phagocyte-pathogen interaction

### 2.1 Intracellular and extracellular microbes

When considering host-directed therapy for bacterial infections, it is important to divide bacteria into those that cause extracellular infections and those that cause intracellular infections. Bacteria that cause extracellular infections include Staphylococcus aureus, Streptococcus pyogenes, Streptococcus pneumoniae, Escherichia coli, Vibrio cholerae, Clostridium tetani, Neisseria meningitidis, and Corynebacterium diphtheriae. On the other hand, bacteria that cause intracellular infections include Mycobacterium, Listeria monocytogenes, and Legionella pneumophila (22). Microorganisms can be classified into three types: 1) obligate extracellular growth parasites, which cannot grow inside the cell but only outside, 2) facultative intracellular growth parasites, which can grow both inside and outside the cell, and 3) obligate intracellular growth parasites, which can grow only inside the cell. Obligate extracellular growth parasites are eliminated by phagocytes and have developed resistance mechanisms against phagocytosis (22). S. aureus is classically recognized as a bacterium that causes only extracellular infections such as furuncles, carbuncles, impetigo, abscesses, septicemia, necrotizing pneumonia, and biofilm formation (28). Recent studies have shown that S. aureus can survive and proliferate intracellularly, which is a major factor in pathogenesis, making it a second category of bacteria (29) (Horn). Bacteria belonging to this category have evolved the ability to neutralize the phagolysosome. The third category of bacteria includes rickettsia and chlamydia, which are dependent on the host in terms of membrane structure and metabolism, respectively, but the immune mechanisms against them are beyond the scope of this review and should be referred to the cited review (30).

Innate immunity to extracellular infections is centered on complement activation, phagocyte activation, and inflammatory responses, and the final execution mechanism of bacterial elimination depends on the phagolysosomal system. Phagocytes directly recognize bacteria via mannose and scavenger receptors and enhance phagocytosis (25). In addition, both peptidoglycan, a major membrane component of Gram-positive bacteria, and LPS, an endotoxin of Gram-negative bacteria, activate the alternative complement pathway to opsonize bacteria. Like complement, bacteria opsonized by antibodies enhance phagocytosis (26). Extracellular bacterial protein antigens cause activation of CD4+ T cells, also assisting phagocytosis. Although neutralization and lysis of bacteria by antibodies are important defense systems, phagolysosomes as the final executor of bacterial elimination are central to bactericidal activity. Bacteria that produce intracellular infections have found a microenvironment (niche) within the phagocyte that is isolated from strong adaptive immunity and have acquired mechanisms that allow them to survive and replicate there. These bacteria have evolved mechanisms to disable the phagolysosomal system within the phagocyte and hijack the phagosome to survive (27). Adaptive immunity attempts to execute bacterial clearance through activation of the phagolysosomal system by recruiting phagocytes with the CD40 ligand signal and INFγ by CD4+ T cells. In the process of escape from the phagosome, the host can trigger a mechanism by which CD8+ T cells, upon receiving the signal, eliminate the infected cell itself (22). This section on host-pathogen interactions describes the general effector function of the host’s phagolysosomal system on pathogens.

### 2.2 From recognition to capture: phagocytosis

**Figure 1:**
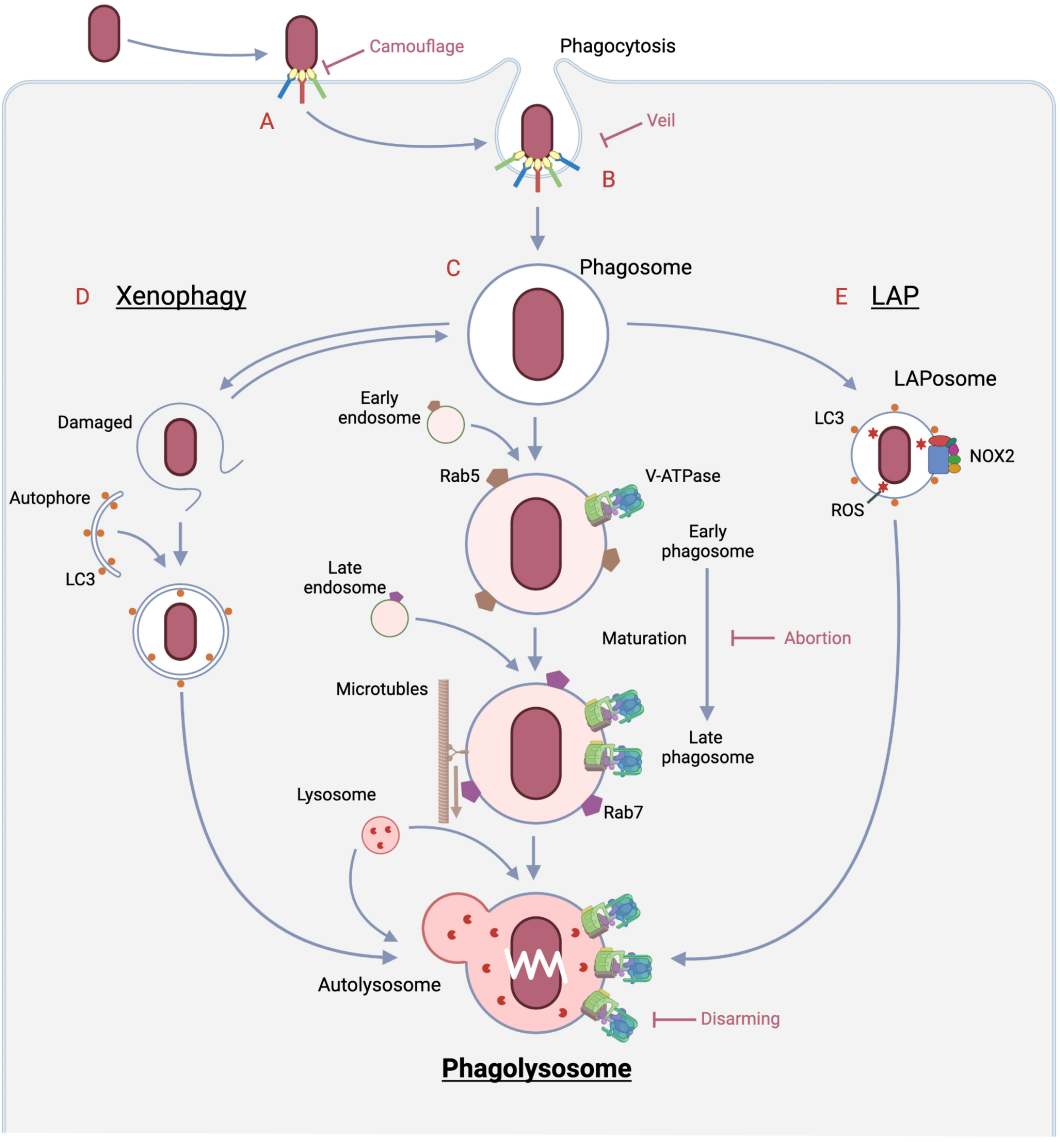
Pathways of bacterial eradication by phagocytes and immune evasion strategies by bacteria. A: The encounter between the bacteria and the host phagocyte is the initial site of rivalry that determines whether infection is established or not, and begins with the sensing of the bacteria by the host. Bacteria LPS is sensed by PPRs, and if opsonized by immunoglobulin and complement, by Fc or complement receptors, respectively. In the case of opsonization by complement, some bacteria evade detection by the complement receptor by mimicking the regulatory mechanism of complement. In the case of opsonization by antibodies, some bacteria prevent binding to the Fc receptor by secreting an enzyme that digests the antibody. Some bacteria alter the structure recognized by PRRs by phosphorylation or other means to escape from the PRRs. B: The process from phagocytic ruffle through cup closure and scission to phagosome formation requires dramatic changes in the cytoskeleton and in membrane phosphatidylinositides, which are regulated by many signals such as phosphorylation. Bacteria prevent phagocytosis formation by disrupting these signals through the secretion of dephosphorylases. On the other hand, there are viruses that produce substances that mimic the “Don’t eat me” signal as phagocytosis checkpoints. C: Phagolysosome pathway (Middle): Nascent phagosomes undergo fusion with early/late endosomes, hydrolase increases toward full set and V-ATPase also increases. As a result, the phagosome lumen becomes acidified and moves along the cytoskeleton toward the site of lysosome presence, where it fuses with the lysosome. The resulting phagolysosome reaches a pH near 4.6, the optimum pH for many hydrolases, to carry out complete bacterial degradation. D: Xenophagy (Left): When the phagosome is damaged by a bacterial escape mechanism, galectin, which is only exposed in the lumen, is exposed in the cytoplasm, which triggers autophagy initiation. If the cargo is a pathogen such as a bacterium, the autophagy is called xenophagy. The vesicles also eventually fuse with the lysosomes, resulting in complete digestion of the pathogen. E: LAP (Right): LAPosomes, in which LC3, which plays an important role in autophagy, engages the nascent phagosome, recruits NOX2 and produces reactive oxygen species. Reactive oxygen species are formed most efficiently in a neutral environment, and they damage pathogen-forming lipids, proteins, and nucleic acids more rapidly than phagolysosomes. The final disposition of the inclusions of this pathway is also completed by fusion with lysosomes. Created in BioRender.

The immune system quickly detects invading bacteria in the body and timely initiates phagocytosis as the appropriate response to eliminate the threat (**Figure 1**) (25). Phagocytes are estimated to make up less than 1% of all cells in the body (31). The ability of these cells to adequately patrol and scavenge throughout the body is critical for defense against foreign enemies (32). Although phagocytes form a constant protrusion (33), and signals from the calcium-sensing receptor (CaSR), a G protein coupled receptor, regulate phosphatidylinositide phosphorylation plasma membrane remodeling (34), and polymerization of the branched actin network just below the plasma membrane (35). Pathogen-sensing receptors include the pattern recognition receptors (PRRs) such as TLR4 that directly bind to pathogen surface structures (24), Fcγ receptors, and complement receptors interact with antibodies or complement that opsonize pathogens, and their interactions play a role in signaling to phagosome formation (25). The pattern recognition receptors (PRRs) involved in bacterial infections are Toll-like receptors (TLRs), nucleotide oligomerization domain (NOD)-like receptors (NLRs), C- and C- type lectin receptors (CLRs), and absent in melanoma-2 (AIM2)-like receptors (ALRs). Ten TLRs have been identified in humans and are present in dimeric form at the plasma membrane or phagosomal membrane (36). In the plasma membrane, TLRs exist as homodimers or heterodimers and recognize lipids, proteins, lipoproteins, and other components of microorganisms. On the other hand, in phagosome membranes, they exist as homodimers and recognize microbial nucleic acids (37). TLR1- TLR2 and TLR2-TLR6 are expressed in monocytes, dendritic cells, and are involved in the recognition of triacyl lipopeptide, lipoprotein, lipopeptide, lipoteichoic acid, arabinomannan, peptidoglycan. TLR4 homodimer is expressed in macrophages and dendritic cells and binds to lipopolysaccharide. TLR4 homodimer is expressed in macrophage and dendritic cells and recognizes lipopolysaccharide. TLR5 homodimer is expressed in intestinal epithelial cells and senses flagellin (24). NOD1 in the cytoplasm of intestinal epithelial cells and macrophages recognizes γ-D-glu-meso-diaminopimelic acid in the cell wall of gram-negative bacteria, while NOD2 recognizes muramyl depeptide in the cell wall of all bacteria (38). CLRs are expressed on macrophages and dendritic cells and play a critical role in anti-fungal immunity. They include the mannose receptor, which recognizes mannose units repeated on the surface of bacteria such as mycobacterium and induces phagocytosis, and the Asian glycoprotein receptor family, which includes Dectin-2, which recognizes mannose-capped lipoarabinomannan (39). ALRs are PRRs that recognize intracellular double strand DNA and do not participate in innate immunity but are involved in apoptosis (40).

The protrusion-captured target induce clustering of phagocytic receptors, and the immunoreceptor tyrosine-based activation motif (ITAM) in their intracellular domain (41) is productively phosphorylated by Src-family tyrosine kinases (SFKs), spleen tyrosine kinases (Syk) (42). As soon as the phagocytosis signal begins to amplify and a transient increase in PI (4, 5)P_2_ occurs, conversion occurs from PI (4, 5)P_2_ to PI (3–5)P_3_ by PI3K recruited to adaptor proteins (43). PI (3–5)P_3_ surges recruits phospholipase Cγ which breaks down PI (3–5)P_3_ to produce diacylglycerol (DAG) and inositol (1, 4, 5)-triphosphate (IP_3_) (44). DAG acts as a second messenger for signaling between phagocytic receptors (45), while IP_3_ provides calcium spike from the endoplasmic reticulum (ER) into the cytoplasm (46). These two 2nd messengers cooperate to activate small G protein Rap1, which mediates the “inside-out” response of integrin (47). PI3K activates Rho family GTPases that facilitate cytoskeletal remodeling directly and through the GEF. This activation dynamically alters the cytoskeleton to form phagocytic cups. NADPH oxidase 2 (NOX2), which is responsible for the generation of reactive oxygen species (ROS) that cause oxidative bursts, engages in the newly formed phagocytic cups (48).

**Figure 2:**
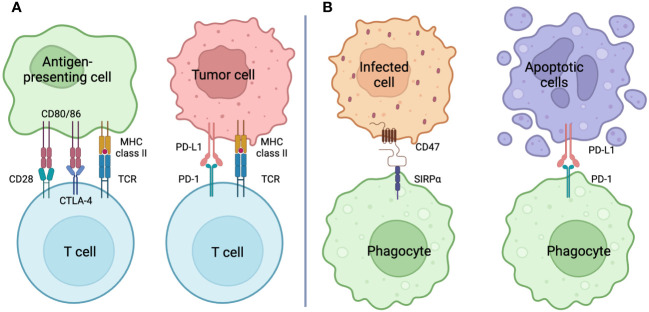
Checkpoint Inhibition **(A)** Immune checkpoint: T cells exercise T cell activation, clonal expansion, and effector function by transmitting signals via the TCR and signals from CD28 through binding to CD80/86 as costimulatory signals. On the other hand, inhibitory signals from CTLA-4 through binding to CD80/86 result in T cell deactivation. T cells expressing PD-1, which is also recognized as a T cell exhaustion marker, transmit inhibitory signals by engaging its ligand PD-L1, causing T cell deactivation similar to that of CTLA-4. Antibodies against molecules that comprise this immune checkpoint (immune checkpoint inhibitors: ICIs) shield the target antigen and cause T cell activation by releasing the T cell brake. Host-directed therapy with ICIs for cancer is an important anti-tumor strategy that works in tandem with anticancer drugs that kill the cancer cells themselves. **(B)** Phagocytosis checkpoint: Phagocytes, like T cells, have receptors with intracellular domains that transmit inhibitory signals to control their activity. The lignads, also called “Don’t eat me” signals, include CD47 and PD-L1, whose receptors are SIRPα and PD-1, respectively. Cells infected with pathogens show enhanced expression of CD47 as the main inducer of INF-g, while cells in apoptosis express PD-L1 and escape clearance by phagocytes. These phagocytosis checkpoint inhibitors may promote phagosytosis and, in the case of bacterial infection, may promote bacterial killing. Created in BioRender.

On the other hand, there are systems that prevent phagocytosis, which phagocytoses pathogens and apoptotic cells, from running amok and eliminating normal cells. In cancer research, immune checkpoints have been identified as entities of T cell regulatory mechanisms (49). Immune checkpoint inhibitors such as anti-programmed cell death protein 1 (PD-1; pembrolizumab and nivolumab) (50), anti-cytotoxic T lymphocyte-associated protein 4 (CTLA-4; ipilimumab and tremelimumab) (51), anti-PD-1 ligand 1 (PD-L1: atezolizumab, avelumab and durvalumab) (52) have developed, and demonstrated to significantly improve outcome in patients suffered from devastating cancers (**Figure 2A**). In innate immunity, phagocytosis checkpoints recognize “Don’t eat me” signals during the phagocytosis process, and are beginning to be recognized as important new targets for cancer immunotherapy (53). The discovery was signal-regulatory protein α (SIRPα) expressed in the myeloid lineage (54). Upon the interaction of SIRPα and CD47, the intracellular domain of SIRPα, an immunoreceptor tyrosine-based inhibitory motif (ITIM) recruits SH2-containing protein tyrosine phosphatase 1 (SHP1) or SHP2, preventing myosin IIA dephosphorylation, subsequent rearrangement of the cytoskeleton, and the phagocytosis they form (55) (**Figure 2B**). Although PD-1 expression is an important marker of T cell exhaustion (56), it also is expressed on various immune cells, including macrophages. The interaction with PD-1 and PD-L1 provides a suppressive signal for the phagocytosis of tumor-associated macrophages (TAMs) (57). Cancer cells can escape macrophage-induced phagocytosis by expressing PD-L1. Sialic acid-binding immunoglobulin-like lectin (SIGLEC), which contains inhibitory receptor motifs (ITIMs) in its intracellular domain, is induced at the surface of macrophages and its expression confers a poor prognosis in cancer patients (58). The ligand for SIGLEC is CD24, and this interaction serves as an entity for anti-phagocytic action (59).

### 2.3 Non-professional phagocytes

Non-specialized phagocytes involved in bacterial infections include epithelial cells, endothelial cells, osteoblasts, and fibroblasts. Epithelial cells, which play another major role among non-specialized phagocytes, cover the outer and luminal surfaces of the body and organs. Depending on where they are present, epithelial cell functions include absorption in the lungs and intestinal tract, secretion in the kidneys and stomach, and material transport in the trachea and oral-nasal cavity. Regardless of their location, the basic function of epithelial cells is to interact closely with the external environment and, in particular, to serve as the first line of defense in the immune system (60). Epithelial cells possess pattern recognition receptors to detect external hazards, but do not express receptors to capture opsonized pathogens as do specialized phagocytes. Therefore, phagocytosis of pathogens by epithelial cells is initiated by two following main methods. One is a trigger mechanism in which the cytoskeleton is restructured by effector molecules secreted by the bacteria, forming ruffles on the plasma membrane, and the other is a zipper mechanism in which the bacteria attach to proteins involved in cell adhesion, such as integrins and cadherins (61). After internalization, the phagolysosome system takes over for clearance.

### 2.4 To destination via 3 routes

Bacterial degradation in first line innate immunity is carried out through three main pathways (**Figure 1**): phagolysosome, xenophagy, and LAP (Microtubule-associated proteins 1A/1B light chain 3B (LC3)-associated phagocytoisis), each of which is followed by a vesicle: phagosome, autophagosomes, and LAPosomes, respectively, through fusion with lysosomes (60). In case of the first pathway, it begins with internalization by phagosomes after pathogen recognition, during which signaling occurs in the cell, leading to phagosome maturation (25) The phagosomes are then translocated to the lysosomes. Subsequently, the pathogen is degraded by phagolysosomes that are generated by fusion with lysosomes (60). On the other hand, autophagy is triggered when the imported bacterium attempts to escape from the phagosome, causing vesicle damage (62). Autophagy to enclose pathogens is called xenophagy, whose efficacy largely depends upon lysosomal function. In the third pathway, LC3, which plays a major role in autophagy, is embedded in the phagosome membrane in the form of LC3 modified by phosphatidylethanolamine (referred to as LC3-II), resulting in the formation of the LAPosome. The LAPosome is partitioned by a single membrane like the phogosome, unlike autophagy, which has a double membrane beginning in the phagophore (63). Its major feature is the quick recruitment of NADPH and the burst of reactive oxygen species that its enzymatic activity leads to, and the maturation of the LAPosome is faster than that of conventional phagocytosis. Review of bacterial killing in LAP and xenophagy are beyond the scope of this review; see other reviews (62, 63).

#### 2.4.1 Phagolysosome pathway: phagosome maturation

Inactivation and decay of phagocytosed pathogens leading to acquired immunity requires dramatic transformation of the formed phagosomes, a process termed phagosome maturation. It is a process that many pathogens target for survival (64). This process leads to two intermediate states: early phagosome and late phagosome, and eventually to the formation of phagolysosomes. Nascent phagosome fuse with early endosomes and are responsible for sorting phagocytosed prey for reusability. The late phagosomes fused with the late endosomes create a more acidic environment in the lumen and migrate along the microtubules toward the lysosomes. The elaborate molecular mechanisms of this process have been detailed, with Rab GTPase and phosphatidylinositide playing major roles.

The newly formed phagosome has a PI (3–5)P3-rich membrane composition, and the recruitment of Rab5 GTPase to it promotes membrane fusion with early endosomes through several pathways (65) Vps34, type III PI3K, is recruited by Rab5 (66) and converts PI to PI3P, which becomes a major component of the membrane and attracts multiple effectors (67). One of them is early endosome antigen 1 (EEA1) (68), which interact with Soluble *N*-ethylmaleimide-Sensitive Factor Attachment Proteins (SNAPs), Syntaxin 6 (69) and Syntaxin 13 (70), to promote membrane fusion of nascent phagosomes and early endosomes (71).

The conversion to the late phagosome begins when the positive feedback loop of Rab5 is severed and replaced by Rab7 (72) PIs that make up the membrane are transformed from PI3P to PI4P by recruitment of 3- phosphatases of the myotubularin family and PtdIns4P kinase 2A (PI4K2A) (73). Rab7 forms homotypic fusion and vacuole protein sorting (HOPS), which mediate a tether between membrane with binding to Rab7, by replacing some of the components of class C core vacuole/endosome tether (CORVET) that mediate Rab5-mediated inter-vesicular tethering (74). GTP-bound active Rab7 recruits two Rab7 effectors: the Rab7-interacting lysosomal protein (RILP) and the long splice variant of the oxysterol-binding protein (OSBP)-related protein 1 (ORP1L) to move toward the microtubule-organizing center (MTOC) for a complete fusion with lysosome (75). They form a scaffold for dynein-dynactin to bridge the microtubule and are transported to the minus end along microtubules (76).

Centripetal movement brings the late phagosome and lysosome into close proximity, and Syntaxin 7 is involved, causing the phagolysosome (77). The two organelles undergo a process of tethering, docking, consolidation, and fusion, in which actin polymerization and calmodulin are involved in the tethering process, protein-protein interaction regulates docking, and Ca spiking leads to consolidation (78). In this stage, the composition of membrane PIs changes dramatically, with PI (3, 5)P2 joining the major PIs components. V-ATPase, which is responsible for intraluminal acidification, is completely incorporated in the phagolysosome membrane (79). Analysis of the immune escape mechanism of *Mycobacterium tuberculosis* revealed that V-ATPase also acts as a major player in membrane fusion and associates with HOPS (80).

#### 2.4.2 Decay

Lysosomes are the defeaters that break down phagocytosed materials into their constituent parts and squeeze out the substances necessary for the host, and they have an arsenal of various weapons for this purpose. Against bacteria, the lysosome attempts to destroy them with reactive radicals, various digestive hydrolases, acidic milieu, and nutrient segregation as its main weapons. However, their regulation differs greatly among cell types, even among professional phagocytes (81). The control of these factors seems to depend on the roles of bactericidal action by reactive oxygen species and nitrogen, followed by complete digestion of the structure and transmission to the acquired immune system by antigen presentation to T cells. Macrophages are highly plastic cells and are classified into two activation states, M1 or classic and M2 or alternative, and the process leading to this state is defined as polarization (82). M1 M2 macrophages are induced by Th1 cytokines such as IFN-γ and TNF-α or LPS and secrete high levels of pro-inflammatory cytokines such as IL-1α, IL-1β, IL-6 and TNF-α. -13 and secrete abundant levels of anti-inflammatory cytokines such as IL-10 and TGF-β (82). Macrophages have been shown to regulate V-ATPase and NOX2 very oppositely by polarization (83). M1 macrophages and neutrophils have low V-ATPase activity and a near-neutral lysosomal lumen but abundant production of reactive oxygen species (ROS). In contrast, M2 macrophages have high V-ATPase activity, the lysosome lumen is strongly acidic, and reactive oxygen species are not so high. However, the classification of M1/M2 macrophages is an oversimplification, as the two states are flexible and dynamically plastic, with intermediate rather than binary states, and there is a subset of regulatory macrophages in addition to activated and healing macrophages (84). It remains to be seen how polarization and its shift are regulated in infection, when the regulatory subset is committed during infection *in vivo*, and what are the keys that control these processes.

##### 2.4.2.1 V-ATPase

**Figure 3:**
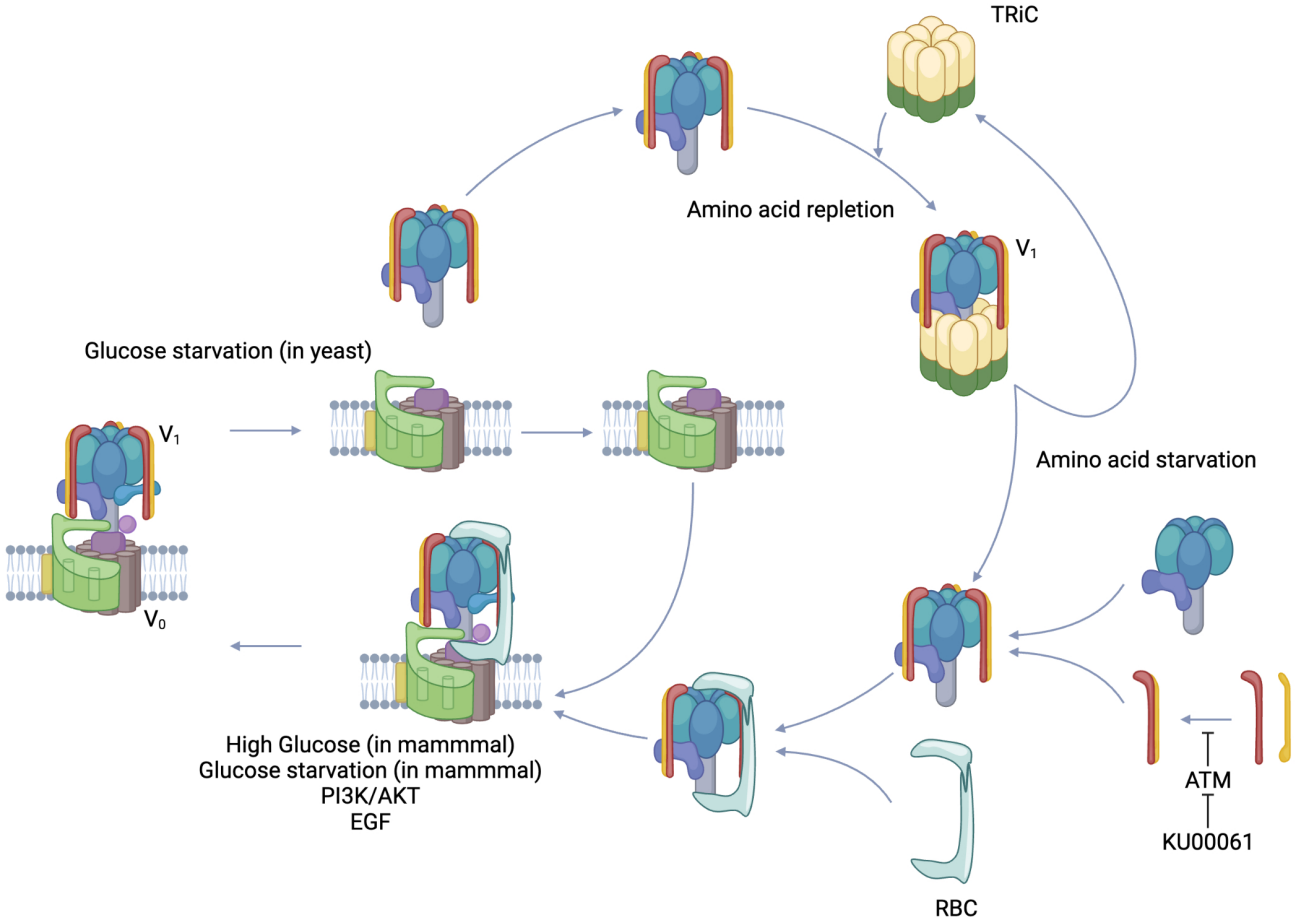
Regulated assembly/Reversible disassembly of V-ATPase. V-ATPase is composed of a membrane-integrated V0 complex and a V1 complex that can exist free in the cytoplasm. PI3K/AKT, EGF signal, and in mammals, glucose starvation and high glucose induce regulated assembly. On the other hand, glucose starvation induces reversible disassembly in yeast. In the case of amino acid deficiency, TRiC, which holds the V1 complex in the cytoplasm, releases the V1 complex and leans toward V-ATPase assembly. Rbc3 recruits V1 to V0 as a chaperone molecule. The dimer formation of V1E and V1G, which form the peripheral stalk of the V1 complex, is inhibited by the phosphorylation of V1G by ATM, resulting in inhibition of V-ATPase assembly. KU-60019, which inhibits ATM, promotes V-ATPase assembly. Created in BioRender.

In lysosomes and endosomes, V-ATPase is the only machinery that consumes energy to transfer protons into the lumen, but the acidic milieu produced by V-ATPase provides an optimal pH for the intraluminal hydrolase to perform bacterial killing (**Figure 3**). V-ATPase also plays an extremely multifaceted role in the phagolysosomal pathway (85). For example, recycling of plasma membrane receptors taken up into the lumen (86), recovery of the mannose 6-phosphate receptor into the trans-Golgi network (87), loading of external antigens into the major histocompatibility complex (88), and endosome tethering in phagosome maturation (80). In addition, acidic milieu plays an essential role in the following processes: neurotransmitter uptake (89), maturation by degradation of prohormone (90), nutrient sensing in association with mTORC1 (91), amino acid supply to the cytoplasm (92), and macroautophagy (93, 94). In signal transduction, WNT and Notch signals require an acidic milieu in the vesicle. In the WNT pathway, the Frizzled/LRP6 complex is in close proximity to V-ATPase via prorenin receptors on the signaling endosome. LRP6 phosphorylation required for the β-catenin destruction inhibition signal is dependent on V-ATPase activity (95). In Notch signaling, the Notch receptor is internalized by ligand binding and transferred onto the signaling endosome, and the acidic environment of the vesicle causes the Notch intracellular domain to be released into the cytoplasm through S3 cleavage by the γ-secretase (96).

Various hydrolases encapsulated in the lysosome have pH-dependent enzymatic activities and their acidity is precisely regulated. The methods reported for its regulation include 1. nutrition, 2. signaling, 3. cofactors, and 4. modification by enzymes. Nutrition was first reported as reversible disassembly in yeast during glucose starvation, and its biological significance is thought to be the limitation of ATP consumption under nutrient-depleted conditions (97). On the other hand, in mammals, V-ATPase assembly was shown to occur at an excess glucose concentration of 25 mM (98). Excess glucose increases glycolysis which leads to acidic environment in the cytoplasm, whereas the promotion of V-ATPase assembly is thought to be responsible for keeping the cytoplasm neutral by accumulating protons in the lysosome. It was reported that reversible disassembly in yeast is mediated by PKA, while regulated assembly in mammals is mediated by PI3K. Initially, the response of V-ATPase to glucose availability in yeast and mammalian cells appeared to be consistent, but the report that glucose starvation also promotes regulated assembly in mammalian cells showed the diversity of the regulatory mechanism (99). Glucose starvation activates AMPK, which is further enhanced by a complex with Regulator and assembled V-ATPase that provides a binding site for AMPK through AXIN (100). This AMPK activation may be directed toward improving energy supply and demand by shifting metabolism toward catabolism, one of which may be autophagy (101). Amino acids also have a significant effect on V-ATPase. In amino acid starvation, Regulator forms a tight complex with V1A of V-ATPase and eliminates mTORC1 while assembling V-ATPase (91). V-ATPase assembly leads to increased activity, and autophagy enhanced by amino acid starvation leads to amino acid acquisition by degradation of proteins brought to the lysosomes, resulting in release of amino acids into the cytoplasm to maintain homeostasis (101). With respect to signals, PI3K and its downstream AKT bring about regulated assembly (102). PI3K inhibitors do not prevent assembly (103), while AKT inhibitors prevent assembly (104). This suggests direct binding of AKT to V-ATPase (105). On the other hand, mTORC1 activity, which is downstream of PI3K/AKT, does not participate in V-ATPase assembly in the absence of amino acids (103). The AKT-mediated increase in the regulated assembly of V-ATPase, which results in decreased intraluminal pH, enhanced proteolysis, and increased cytoplasmic amino acid content, is also the mechanism by which EGF activates mTORC1 (106).

Cofactors and assembly chaperones such as Rabconnectin3 (Rbc3), TRiC, and mEAK7 and enzymes such as ATM have been reported. In yeast, RAVE (Regulator of H+-ATPase of Vacuolar and Endosomal membranes) regulates luminal acidity of lysosomes and endosomes via V-ATPase assembly, and in mammals Rbc3 is functionally equivalent to RAVE (107). Rbc3 is a heterodimer composed of Rbc3α and Rbcβ (108). The former is composed of either of two isoforms, DMXL1, or DMXL2 (109), and the latter is formed by WDR7. The combination varies among tissues and intracellular organelles (107). Rav1, which is a subunit of the yeast RAVE, recruits free V1C in the cytoplasm and contributes to V-ATPase assembly (110). DMXL1 and DMXL2 are homologs of Rav1, and the amino acid sequence in which Rav1 interacts with V1C is also conserved in DMXL (111). Functionally, silencing of any of the components of Rbc3 reduced the acidity in the vesicles (112). Knockout of WDR7 attenuated V-ATPase assembly (113). The regulation of Rbc3 is still to be elucidated, but one clue is calcium dynamics. CAB2.2, a transmembrane calcium channel, binds to DMXL (114), and CAPS1, which is involved in endoplasmic reticulum acidification through calcium dynamics, also binds to Rbc3 (115). TRiC holds the V1 component in the amino acid-replete cytoplasm, while releasing it for V-ATPase assembly in the presence of amino acid deprivation (116). A regulatory mechanism of TRiC could the phosphorylation of a subunit constituting TRiC. Phosphorylation of CCT2, a component of TRiC, modulates its function (117). mTORC1 signal may modify TRiC components to stabilize the TRiC/V1 component complex. mEAK7 engages V1A, B, and E in the N-terminal domain and binds to V1D in the C-terminal domain, but does not contribute to luminal acidification and affects mTOR signaling (118). Although ataxia telangiectasia mutated (ATM) was initially identified as a protein involved in the DNA damage response, it was recently reported to phosphorylate V1G and prevent the interaction with V1E, resulting in inhibition of the formation of a peripheral stalk (119).

##### 2.4.2.2 Reactive radicals

Among the microbicidal effects induced by bacterial phagocytosis, the production of reactive radicals in the lumen is mainly mediated by NOX2 of the NADPH oxidase (NOX) family and inducible nitric oxide synthase (iNOS), which are triggered most rapidly after pathogen entry. The former produces reactive oxygen species (ROS) most prominently in neutrophils, while the latter produces reactive nitrogen species (RNS) mainly in macrophages (64). The superoxide anion (O_2_^-.^) produced by NOX2 leads to the production of ROS represented by hydrogen peroxide (H_2_O_2_), hydroxyl radical (OH*), and hypochlorous acid (HOCl). This process is called a respiratory or oxidative burst because of the surge in oxygen uptake and glucose consumption unresponsive to cyanide (120).

The activated NOX2 complex transfers electrons from the cytoplasmic NADPH into the lumen of the phagosome, and the resulting charge imbalance is resolved by the voltage-gated proton channel Hv1, indicating that the activity of the NOX2 complex requires this channel to be activated (121). Since this ion channel does not consume energy, it is thought to function only when the pH of the phagosome lumen is near neutral, indicating that the respiratory burst of phagocytes induced by NOX2 complex activation occurs only in a very narrow range near neutral (122). The superoxide anion (O_2_^-^) generated by the NOX2 complex can utilize electrons from further NOX2 complexes and hydrogen from the phagosome lumen and Hv1 to induce superoxide reductase (SOR) or three types of superoxide dismutases (SODs) to produce hydrogen peroxide, leading to the formation of additional hydroxy radicals (123). These reactive radicals carry out their microbicidal action by disrupting structures containing DNA, Fe-S clusters, hemes, sulfhydryls, thioethers, and alkenes (124). ROS from the NOX2 complex are not prominent in macrophages, but in macrophages that swallowed the pathogen in defense against *S. aureus* infection, mitochondria-derived vesicles, which contain abundant hydrogen peroxide, fuse with phagosomes to provide reactive radicals, which are lacking in bacterial killing (125).

It is mainly the inducible nitric oxide synthase (iNOS) that produces reactive nitrogen species (RNS) that cooperate with ROS in pathogen killing (64). Nitrogen oxide is made of cytoplasmic L-arginine and oxygen, which undergo various catalytic reactions to produce nitrogen dioxide, peroxynitrite, dinitrogen trioxide, and dinitrosyl iron. Unlike NOX2, the regulatory mechanism occurs at the transcriptional regulation, and *de novo* protein synthesis is required for RNS production (126). Activation signals for iNOS include the extracellular proinflammatory cytokine interferon gamma (IFNγ) and the intracellular signaling molecule NF-κB (127).. It had been thought that iNOS is not recruited to the phagosome and remains in the cytoplasm; therefore, the RNS produced reaches the phagosome lumen by diffusion (128). In research on *Mycobacterium* spp., it was revealed that iNOS is recruited to phagosomes through binding with the scaffolding protein EBP50, while the bacillus attenuates the recruitment (129).

##### 2.4.2.3 Nutrients

Iron, alone or incorporated into Fe-S clusters or heme, is essential for respiration, amino acid metabolism, and nucleic acid synthesis, not only in eukaryotes but also in prokaryotes. Excess iron leads to the ROS formation, while catalases and peroxidases that relieve oxidative stress require heme as a cofactor (130). The innate immune system has acquired the tactic of making iron unavailable to pathogens so that they can feed on the pathogens they have taken in (131). Lactoferrin is structurally very similar to transferrin, and it strongly binds to divalent iron ions even in the highly acidic environment of the lumen of the lysosome and exhibits antimicrobial action as an iron chelating agent (132). Iron is absorbed by bacteria via siderophore from the environment. Siderocalin (neutrophil gelatinase-associated lipocalin (GAL)), which inhibits siderophores, has been shown to effectively function, especially in sepsis caused by *E. coli (* 133) and *Mycobacterium* spp (134).. Natural resistance-associated macrophage protein-1 (Nramp1/Slc11a1), in the membrane of phagosomes and functions as a divalent metal-proton symporter, has been implicated in the defense of intravesicular pathogens (IPs). Nramp1 starves IPs such as *Mycobacteria, Salmonella typhimurium*, and *Leishmania domovanii by* removing Fe2+, Co2+, and Mn2+ from the phagosome (135).

## 3 Phagocyte-pathogen interaction: evasion 

**Figure 4:**
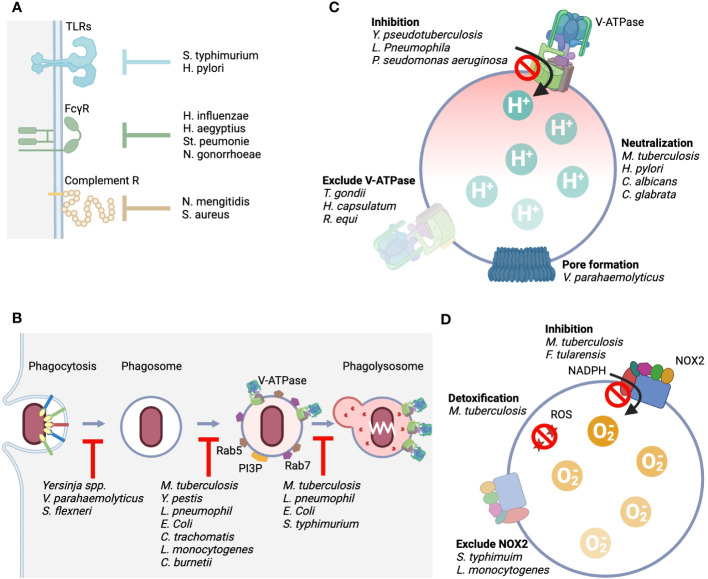
Rpresentative mechanisms by which pathogens evade from the host phagolysosomal system. **(A)** Representative mechanisms by which the host senses pathogens include TLRs, FcγRs, and complement receptors, and we show bacteria that possess mechanisms that counteract this sensing. **(B)**. It shows bacteria with an evade mechanism by inhibiting the process of phagocytosis, phagosome maturation, and fusion with lysosomes. **(C)**. The point of action at which the function of V-ATPase is attacked and the bacteria that carry it out. Four typical mechanisms are shown. Direct inhibition of pump function, elimination of the pump from the membrane, loss of proton concentration differences by generating pores, and production of enzymes that alkalinize acidified intralumens. **(D)**. The point of action at which the function of NOX2 is attacked and the bacteria that carry it out. Three typical mechanisms are shown: direct inhibition of the mechanism that produces superoxide, elimination of the complex from the membrane, and detoxification of the produced reactive oxygen species.

Pathogens have various strategies to evade immunity and survive. In particular, the pathway from phagocytosis to digestion in the phagocyte, the first line of defense of innate immunity that the pathogen encounters, is the most important site that must be neutralized (64). Numerous molecular mechanisms have been described that allow pathogens to disarm the phagosome pathway and thereby acquire the microenvironment in which to survive and proliferate (**Figure 4**; **Tables 1**–**3**) (136).

**Table 1:** Immune evasion strategies for processes from sensing to internalization.

Host process	Pathogen	Effectors	Mechanism
**Sensing**	*Salmonella typhimurium*	Lipid A: Deacylation/Palmitoylation	Escape from TLR4
	*Helicobactor pylori*	Lipid A: Dephosphorylation	Escape from TLR4
	*Haemophilus influenzae*	Proteinase for IgA	Inhibit the binding to FcgR
	*Haemophilus. aegyptius*	Proteinase for IgA	Inhibit the binding to FcgR
	*Streptococcous peumonie*	Proteinase for IgA	Inhibit the binding to FcgR
	*Neisseria gonorrhoeae*	Proteinase for IgA	Inhibit the binding to FcgR
	*Neisseria mengitidis*	Recruit complement factor H (fH)	Inhibit the binding to CR
	*Staphylococcus aureus*	SdrE to recruit fH	Inhibit the binding to CR
**Phagocytosis**	*Yersinia* spp.	protein tyrosin phosphatase: YopH	Inhibit the capturing
	*Vibrio parahaemolyticus*	VPA0450: inositol polyphosphate 5-phosphatase	Blebbing
	*Shigella flexneri*	IpgD: inositol 4-phosphatase	Blebbing

**Table 2:** Immune evasion strategies to prevent phagosome maturation.

*Mycobacterium tuberculosis*	NdkA as small GTPase inhibitor	Inhibit RAB5 and RAB7
	SapM as PI3P phosphatase	Inhibit PI3P generation
	MptpB as PI3P, PI4P and PI5P phosphatase	Arrest phagosome maturation
	ManLAM to activate calcium-dependent calmodulin	Inhibit PI3P generation
	phosphatidylinositol mannoside(PIM)	Inhibit RAB7
	trehalose dimycolate (TDM)	Inhibit lysosomal fusion
	sulfoglycolipid-1(SL-1)	Inhibit lysosomal fusion
	protein tyrosine phosphatase A (PtpA)	Inhibit lysosomal fusion
*Yersinia pestis*	Recruit RAB4a and RAB11b	Deviate the recycle
	Recruit RAB1b	Inhibit maturation
*Legionella pneumophil*	Dot/Icm type IV secretion system	Over 330 biological process affected
	SidM/DrrA: recrut RABa	Inhibit endosome fusion
*Escherichia. coli*	K1 capsule (α-2,8-kinked polysialic acid)	Inhibit lysosomal fusion
	Tir as a scaffold to SHIP2	Induce actin pedestal formation
*Salmonella typhimurium*	SopB as PI (4,5)P2 phosphatase	Inhibit lysosomal fusion
*Chlamydia trachomatis*	Not determined	Deviate to the secretary path
*Listeria monocytogenes*	Listeriolysin O	Generate pores in phogosomal membrane, leading to escape to cytosol
	Phospholipase (PlcA)	Purturb phagosomal membrane
	Phospholipase (PlcB)	Purturb phagosomal membrane
*Coxiella burnetii*	Ank as a type IV secretion system protein	Delayed maturation

**Table 3:** Immune evasion strategies to neutralize bacterial destruction mechanisms.

Host process	Pathogen	Effectors	Mechanism
**V-ATPase**	*Mycobacterium tuberculosis*	Protein tyrosine phosphatase A (PtpA)	Inhibit lysosomal fusion
		Antacid 1-tuberculosinyladenosine (1-TbAd)	Neutralization
	*Helicobacter pylori*	Urease	Neutralization
	*Candida albicans*	Urease	Neutralization
	*Candida glabrata*	Urease	Neutralization
	*Rhodococcus equi*	Virulence-associated protein A (VapA)	Exclude V-ATPae
	*Histoplasma capsulatum*	Not identified	Exclude V-ATPae
	*Yersinia pseudotuberculosis*	Not identified	Inhibit proton pump
	*L. pneumophila*	SidK to bind V1A	Inhibit proton pump
	*Pseudomonas aeruginosa*	pyocyamin	Inhibit proton pump
	*Vibrio parahaemolyticus*	VopQ	Neutralization by pore formation
	*Toxoplasma gondii*	Not identified	Exclude V-ATPase
**Reactive radicals**	*Salmonella typhimuium*	Salmonella pathogenicity island-2 (SPI2)	Inhibit the accumulation of flavocytochrome b558
	*Listeria monocytogenes*	Pore-forming cytolysin listeriolysin O	Exclude NOX2
	*Fransicella tularensis*	fevR	Inhibit NOX2 activity
	*Mycobacterium tuberculosis*	Iron-dependent enzyme (SodA)	Detoxification
		Copper/zinc-dependent enzyme (SodC)	Detoxification
		KatG: Catalase/Peroxidase/Peroxynitritase	Detoxification
		CpsA	Inhibit NOX2 activity

### 3.1 Camouflage/veil

*Salmonella typhimurium* interferes with TLR4 recognition and signaling through deacylation and palmitoylation of lipid A present on their surfaces (137). *Helicobacter pylori* dephosphorylates lipid A to escape TLR4 (138). *Haemophilus influenzae, H. aegyptius, Streptococcus peumonie*, and *Neisseria gonorrhoeae* secrete proteases that selectively cleave immunoglobulin A, which is responsible for opsonization (139, 140). Complement also plays a role in opsonization, but some bacteria exploit the elaborate activation pathway of complement. *Neisseria mengitidis* is a bacterium that avoids phagocytosis by mimicking the host complement factor H (fH), the regulatory substance of complement, on its own surface (141). *Staphylococcus aureus* also recruits fH to its surface by secreting a substance called SdrE (142). YopH, a protein tyrosine phosphatase produced by *Yersinia* spp., dephosphorylates host phosphotyrosine proteins and prevents phagocytosis (143). *Vibrio parahaemolyticus* secretes an inositol polyphosphate 5-phosphatase, VPA0450, which disrupts host cell membrane integrity and causes blebbing (144). This tactic is *also used by Shigella flexneri, which* secretes IpgD, an inositol 4-phosphatase, as its virulence factor, causing plasma membrane blebbing by converting PI (4, 5)P2 to PI (5)P (145). Although not a bacterium, m128L encoded by *Myxoma virus* has a high homology to CD47, which is known to inhibit host phagocytosis as a “don’t eat me” signal (53). The “don’t eat me” signal is essential for establishing the lethal infection through inhibition of host phagocytosis (146). Although there are no reports of cases in which the bacteria themselves encode CD47 mimic, enhanced expression of CD47 in cells infected with *S. typhi* and *Borrelia burgdorferi* has been reported (147). The upregulation of CD47 expression occurs through signaling from PRRs and is also enhanced by inflammatory cytokines, suggesting that the CD47-SIRPα axis may work to suppress excessive inflammatory responses. Bacteria make good use of this host regulation mechanism to aid immune evasion. On the other hand, *M. tuberculosis*, a phagocytosis-dependent intracellular parasite, is unique in that it does not enhance CD47 expression, unlike other bacteria.

### 3.2 Neutralization of intracellular microbial killing machineries

Although we are far from having a complete picture of the diverse strategies by which bacteria neutralize host immune attack within the cell, learning the molecular mechanisms is the first thing we must do to win the war against bacteria. The strategy of bacteria that acquire permissive niches intracellularly as intracellular pathogens (IPs) (148) provides a great clue for the construction of host-directed therapeutics (HDTs) (27). *M. tuberculosis*, a leading IP, has long been a major scourge to mankind due to its high prevalence and high mortality rate on a global scale (149), and is one of the most carefully investigated (150). *M. tuberculosis* is thought to have evolved in such a way to struggle with the host immune system that an exhaustive list of protein and lipid effectors produced by the bacillus has been compiled (151). In addition to *M. tuberculosis*, other potential IPs include *Rickettsia rickettsia, Chlamydia trachomatis, Legionella peumophila, Coxiella burnetiid, Brucella abortus*, and *Salmonella enterica*; *Cryptococcus neoformans and Aspergillus fumigatus* among fungi. *Candida albicans* uses other intracellular organelles such as mitochondria as a habitat. The mechanisms by which they evade the phagosome pathway have been intensively studied, and detailed molecular mechanisms have been elucidated (152).

#### 3.2.1 Abortions of phagosome

*M. tuberculosis* has spun out more than a dozen countermeasures in the phagosome maturation stage alone. First, it secretes NdkA to repress the small GTPases RAB5 and RAB7, which are central regulatory molecules in phagosome maturation (153). In addition, SapM produced by the bacillus acts as a PI3P phosphatase and inhibits the formation of PI3P, which is essential for the maturation of membrane composition (154). Glycolipids on the surface of the bacillus also greatly influence this phagosome maturation. Mannose-capped lipoarabinomannane (ManLAM) suppresses PI3P generation via the calcium-dependent calmodulin pathway and prevents phagosome maturation (155). In addition, phosphatidylinositol mannoside (PIM) present in the envelope supports the retention of early endosome RAB proteins such as RAB5, RAB22A, and RAB14 and prevents the recruitment of late endosome RAB proteins such as RAB7 (156). With respect to fusion with the lysosome, trehalose dimycolate (TDM) (157) and sulfoglycolipid-1 (SL-1) (158) as lipid effectors prevent the fusion process between the lysosome and the phagosome. Furthermore, *M. tuberculosis* secretes toxins that inhibit the Ca/Calmodulin-PI3K cascade and attempts to survive as IP through a strategy of inhibiting the fusion of phagosomes and lysosomes (159). In phagosomes harboring *M. tuberculosis*, protein tyrosine phosphatase A (PtpA) secreted by the bacillus binds to V-ATPase V1H and inhibits the association of V-ATPase and HOPS, as well as dephosphorylates vacuolar protein sorting 33B (VPS33B), which forms HOPS, and thus loses its function as a fusion machinery (80).

*Yersinia pestis* targets organelle trafficking and recruits Rab4a early in infection and Rab11b late in infection to prevent phagosome maturation and inhibit acidification in the lumen (160). These small GTPases are involved in endosome recycling, and the *Yersinia*-containing vacuole mimics this process. In addition, *Y. pestis* recruits Rab1b to phagosomes to inhibit phagosome acidification by suppressing lysosome fusion (161). *Legionella pneumophila* has evolved a defect in organelle trafficking: intracellular multiplication (Dot/Icm) type IV secretion system to make the phagosome of alveolar macrophages a proliferative niche (162). This system provides more than 330 effector proteins that interfere with host biological processes to assist in bacterial replication and survival (163). Among them, the system is involved in the recruitment of Rab1 like *Y. pestis (* 164) and provides Sid1/DrrA, which is involved in the regulation of Rab1 (165). *E.coli* K1 has a K1 capsule composed of α-2,8-kinked polysialic acid on its surface that inhibits the fusion of phagosomes and lysosomes. *Salmonella*-containing phagosomes also inhibits the fusion of phagosomes and lysosomes (166). *Salmonella* secretes phosphoinositide phosphatase to maintain PI3P levels in the membrane, thereby preventing phagosome maturation and fusion with the lysosome and ensuring its survival (167). *Chlamydia trachomatis* avoids the fusion of its internalized phagosomes with endosomes and directs them to the secretory pathway to avoid an acidic environment (168).

#### 3.2.2 Disarming V-ATPase

It was shown 30 years ago that *M. tuberculosis*, when it reaches the phagolysosome, excludes V-ATPase from its membrane and maintains the lumen at a pH of 6.3 or higher (169). The entity responsible for excluding V-ATPase from the phagosome was PtpA (80). In addition, antacid 1-tuberculosinyladenosine (1-TbAd), which neutralizes acidification of the lumen, is secreted by the bachillus (170). *H. pylori, which* can live in highly acidic stomachs, has evolved various genes to adapt to the acidic environment. One of the effectors is urease, which produces ammonium ions that allow the pathogen survive in the harsh acid environment of the stomach (171). *Candida albicans and C. glabrata* use amino acids in their lysosomes to produce ammonium ions to neutralize the intraluminal pH (172, 173). *Mycobacteria* spp. have the same strategy (174). Research has been conducted to create more effective vaccine that lacks urease (175). a single bacterium has multiple defense mechanisms against the host offense of lysosomal acidification. In addition to *Mycobacterium* spp., *Rhodococcus* spp (176, 177). and *Histoplasma capsulatum (* 178) were reported to exclude V-ATPases from phagosomes that contain them.

Some bacteria secrete substances that directly inhibit V-ATPase. *Y. pseudotuberculosis* directly inhibits the activity of the proton pump without affecting the expression level of the protein component of V-ATPase, thereby causing lysosomal deacidification (179). SidK produced by *L. pneumophila* physically binds to V-ATPase V1A and inhibits its proton transport (180). Structural analysis of the binding of SidK to V-ATPase showed that the two α-helical bundles at the N-terminus of SidK bind to V1A and markedly reduce the flexibility of its subunit (181). *Pseudomonas aeruginosa* secretes pyocyamin, which is a potent inhibitor of V-ATPase (182). *Toxoplasma gondii* survives by eliminating all components involved in membrane fusion with endosomes, resulting in non-acidic vacuole (183, 184). *Vibrio parahaemolyticus* secretes VopQ, a type III effector protein, which is incorporated into the lysosome membrane as a channel for the free passage of protons, and the pH in the cytoplasm and lysosome lumen is balanced (185).

#### 3.2.3 Disarming NOX2

*Salmonella typhimurium* inhibits the accumulation of flavocytochrome *b*_558_ by releasing Salmonella pathogenicity island-2 (SPI2), a member of the type III secretion system (186). *Listeria monocytogenes* also eliminates the NOX2 membrane component by secreting the pore-forming cytolysin listeriolysin O (187). *Fransicella tularensis* not only excludes flavocytochrome *b*_558_ but also directly inhibits the activity of the NOX2 complex by releasing a regulatory factor called fevR (188). *M. tuberculosis* has also taken multiple countermeasures against reactive radicals, including two types of SODs that process ROS: iron-dependent enzyme (SodA) (189) and copper/zinc-dependent enzyme (SodC) (190) and both contribute significantly to the virulence of the pathogen. In addition, the bacillus secretes KatG, which serves as a peroxidase and peroxynitritase, to metabolizes reactive radicals produced by the phagocyte oxidative burst (191). When the bacillus is preyed upon by the LAPosome, it secretes CpsA as an effector and inhibits the activity of NOX2 (192).

#### 3.2.4 Securing nutrition

Gram-negative rods *such as E. coli, Salmonella* spp., and *Klebsiella pneumoniae* restore the host-inhibited function of their own siderophores, which are responsible for iron absorption, by producing a protein called iroA (193). The thick waxy cell walls of *Mycobacterium* spp. provide excellent protection against severe environmental and host invasion, but are not conducive to the exchange of nutrients and metabolites necessary for growth and survival with the outside world. *Mycobacterium* attempts to secure iron by producing mycobactin as a siderophore (194). The host interferes with mycobactin, as it did against siderospheres of gram-negative rods, but *Mycobacterium* secretes Esx-3 of the type VII secretion system (Esx-1-5) to support mycobactin and iron absorption (195). *Aspergillus fumigatus* produces HapX in iron deficiency to suppress iron-consuming pathways such as host heme synthesis and respiration, including the TCA circuit, and to increase the production of iron-absorbing siderophores (196).

## 4 Host-directed therapy for bacterial infections

Against bacterial infections, tremendous resources have been devoted to the development of antimicrobial agents that kill the bacteria themselves. Cancer therapy has long since moved beyond the days when drugs were developed to kill cancer cells themselves, and HDTs have become a major pillar of cancer treatment. The development of HDTs for bacterial infections has just begun, and the development of HDTs for the phagolysosome pathway, the first line of innate immunity, has lagged further behind. The molecular mechanisms of the phagolysosome and bacterial evasion strategies described thus far provide major clues to HDT. In the following part, we would like to describe the current status of drug discovery that intervenes in the phagolysosome pathway.

### 4.1 Phagocytosis activator

The application of immune checkpoint blockade to infectious diseases has been investigated in the context of the interrelationship between innate and adaptive immunity, rather than between infected cells and innate immunity. Interventions on the PD1-PD-L1 axis are effective in animal studies against infectious diseases such as *malaria, toxoplasma, leishmania, and Listeria (* 197). The results of a phase 1/2 trial of nivolumab in sepsis have overcome safety concerns, including the development of autoimmune disease (198). In cancer therapy, although macrophages have been intensively studied as targets for intervention in various aspects, phagocytosis that macrophages execute is recognized as a promising drug discovery (199). The findings of the phagocytosis checkpoint may provide clues to the treatment of infectious diseases (53). The inhibition of the PD-1-PD-L1 axis as a phagocytosis checkpoint enhances phagocytosis in liver Kupffer cells and prevent bacterial infection (200). A study using CD47 KO mice also reported that *E. coli* pneumonia showed better recovery compared to wild type (201).

### 4.2 V-ATPase activator

It was reported that monocytes from imatinib-treated patients with leukemia showed an increased production of V0a3 and V0c and had more acidic lysosomes. Sera from those patients, which are added to the cell culture of macrophages, enforced more acidic lysosomes and of *M. tuberculosis (* 202). One compound was reported to promote regulated assembly of V-ATPase, KU-60119, which was identified as an ATM inhibitor that inhibited assembly of V1E and V1G through phosphorylation of the latter (119). ZLN005 was originally recognized as a peroxisome proliferator-activated receptor gamma coactivator 1-α (PGC1α) activator (203). By administering ZLN005 to the cecum perforation ligation (CPL) model of sepsis and analyzing intraperitoneal cells, ZLN005 was shown to be a transcription factor EB (TFEB) activator involved in lysosome biogenesis as well as a lysosomal acidifier (204). The compound significantly improved overall survival and drastically reduced intraperitoneal bacterial load in mice at only 2 h after administration of ZLN005 in *in vivo* sepsis model. These results indicate that lysosomal acidification is a therapeutic target for sepsis.

## 5 Clinical aspects and limitations in host-directed therapy

When considering the pathogenesis of sepsis (205), in most cases the organism of origin is not known at the onset of the disease. When the pathogen is unknown, the choice of treatment, especially antimicrobial agents, is highly dependent on the experience and ability of the clinician. In contrast, the existence of host-directed therapies that are independent of the organisms causing the disease can first reduce the bacterial load. Even if not eradication, significant improvement in survival may be achieved if circulatory collapse and multiorgan damage can be avoided by reducing the excessive bacterial load to a controllable level, which has been beyond the reach of current initial therapy. When addressing infections caused by resistant bacteria (206), countering bacterial interference with lysosomes, which play a crucial role in bacterial elimination, emerges as a significant alternative to traditional disinfection methods.

The disadvantage of augmenting the phagolysosomal system is that overactivation of autophagy is a concern, since the lysosomal system is the final point of autophagy, which forms the basis of cellular function (62). There is also concern that digestion in lysosomes may be problematic by causing energy depletion in the cell, as significant energy expenditure is thought to occur during digestion in lysosomes (20). These are all reasons to believe that if host-directed therapeutics were to be discovered, the method of administration would need to be carefully set up.

## 6 Perspective

Bacterial infections are a major public health threat no less than malignancies, neurodegenerative diseases, and cardiovascular and metabolic diseases. The development of antibiotics with the goal of disinfection shines as the most significant achievement of 20th century medicine (2). Now, the development of new antibiotics against the emergence of resistant bacteria is taking a backseat to bacterial evolution. Anticancer therapy has seen a breakthrough with the establishment of host-directed therapy in parallel with the development of therapeutic agents aimed at eliminating cancer cells (207). Host immunity plays a major role in the pathogenesis of both cancer and infectious diseases (197). However, host-directed therapy for infectious diseases is still in its infancy (208). In both innate and adaptive immunity, the phagolysosomal system plays a central role in bacterial clearance against bacterial infections (22). Bacteria have evolved to achieve evasion of this phagolysosome system by any means necessary (64, 183). Due to rapid advances in antibiotic technology, the host has not yet evolved to acquire an effector mechanism to counter the evasion mechanism exhibited by resistant bacteria. On the other hand, detailed molecular biological analyses of this system have been performed (26, 101, 209), and the time is ripe for the development of drugs with a point of action in this system. Possible targets of action are numerous, including phagocytosis, phagosome maturation, fusion with lysosomes, lysosome acidification, and lysosome quality control. This system is a fundamental cellular system and is also heavily involved in neurodegenerative diseases (93) and embodies the development strategy of organelle drug discovery rather than the framework of disease-by-disease drug discovery. The development of host-directed therapeutics for bacterial infections has the potential to revolutionize the drug discovery system. It is hoped that the development of HDT together with a new class of antimicrobial agents, will work like two wheels on a cart, dramatically increasing the life-saving rate of sepsis and creating a treatment strategy that is not afraid of superbugs.

## Author contributions

SG designed the concept of this review. SG and TT wrote and edited the manuscript and the table. FT prepared figures. All authors contributed to the article and approved the submitted version.
